# Epidemiology and Investigation of Melioidosis, Southern Arizona

**DOI:** 10.3201/eid1707.100661

**Published:** 2011-07

**Authors:** Tasha Stewart, David M. Engelthaler, David D. Blaney, Apichai Tuanyok, Eric Wangsness, Theresa L. Smith, Talima Pearson, Kenneth K. Komatsu, Paul Keim, Bart J. Currie, Craig Levy, Rebecca Sunenshine

**Affiliations:** Author affiliations: Arizona Department of Health Services, Phoenix, Arizona, USA (T. Stewart, E. Wangsness, K.K. Komatsu, C. Levy, R. Sunenshine);; Translational Genomics Research Institute, Flagstaff, Arizona, USA (D.M. Engelthaler, P. Keim);; Centers for Disease Control and Prevention, Atlanta, Georgia, USA (D.D. Blaney, T.L. Smith, R. Sunenshine);; Northern Arizona University, Flagstaff (A. Tuanyok, T. Pearson, P. Keim);; Menzies School of Health Research and Royal Darwin Hospital, Darwin, Northern Territory, Australia (B.J. Currie)

**Keywords:** melioidosis, Burkholderia pseudomallei, gram-negative bacterial infections, zoonoses, Arizona, dispatch

## Abstract

*Burkholderia pseudomallei *is a bacterium endemic to Southeast Asia and northern Australia, but it has not been found to occur endemically in the United States. We report an ostensibly autochthonous case of melioidosis in the United States. Despite an extensive investigation, the source of exposure was not identified.

*Burkholderia* *pseudomallei* is endemic to Southeast Asia and northern Australia; the organism has also been identified on other continents and islands but not North America (*1*). *B. pseudomallei *is present in soil and water and can cause infection through inhalation, aspiration, ingestion, or percutaneous inoculation (*2**–**4*). Persons with certain chronic health conditions, particularly diabetes, are predisposed to melioidosis disease after exposure to this bacterium. Person-to-person transmission has been documented but is rare (*5*). Clinical signs of the disease vary, depending in part on the route of exposure, and can manifest as pneumonia, septicemia, or single or multiple abscesses (*2*). Treatment is prolonged and made more difficult by the bacterium’s intrinsic resistance to antimicrobial drugs (*6*). No cases of *B. pseudomallei* infection have been documented in the United States in persons without a history of prior travel to a country where the disease is endemic (*7*).

## The Study

In October 2008, a 32-year-old man with a history of type II diabetes, hypertension, and obesity was admitted to a small community hospital (hospital A) in Arizona. He had severe right knee pain and fever. On 3 occasions the week before being admitted, he had been evaluated in hospital A’s emergency department for severe right knee pain. The patient denied cough, chest pain, and swelling and pain of other joints. Over the course of 2–3 months, he lost weight and had exhaustion, intermittent right knee pain, and nightly fevers. The patient denied recent trauma.

Medications at admission included those common for control of diabetes. The patient did not smoke, drink alcohol, or use illicit drugs and was in a monogamous heterosexual relationship. He worked at an automobile body shop preparing and painting cars and had previously worked as a motorcycle and all-terrain vehicle mechanic. He reported gardening as a hobby and had many indoor and outdoor plants. He also had several dogs in the household but reported no unusual exposure to other animals.

After admission to hospital A, the patient underwent arthrocentesis of the right knee. Culture of the synovial fluid did not yield any bacterial growth, and evidence of infection or crystals was not apparent. A blood specimen was sent for culture and yielded what was initially identified as *Escherichia coli* by an automated in-house instrument. Sensitivity to antimicrobial drugs was consistent with this bacterium.

Chest radiograph and computerized axial tomography scan with contrast were unremarkable and did not demonstrate evidence of pneumonia. Laboratory tests indicated no sexually transmitted infections. The patient received clindamycin, imipenem, vancomycin, and metronidazole intravenously. After 8 days in hospital A and no resolution of fever or knee pain, he was transferred to hospital B, a large regional hospital, with an initial diagnosis of persistent *E. coli* sepsis and possible vegetative valve lesions. Transesophageal echocardiogram performed at hospital B did not show vegetative valve lesions.

After admission to hospital B, the patient underwent arthrocentesis of the right knee. Although there was evidence of infection, synovial fluid culture yielded no growth. Blood cultures grew *B. pseudomallei* identified by an automated in-house instrument. Because initial results were unexpected, blood samples were submitted to a reference laboratory and the Arizona State Public Health Laboratory (Phoenix, AZ, USA) where results confirmed the presence of *B. pseudomallei*. The bacteria continued to grow in blood cultures for 16 days after initial hospitalization at hospital A on October 7. Cultures of the knee fluid grew *B. pseudomallei* for 7 days; sputum cultures were positive for 6 days. All cultures were negative 2 weeks before the patient was discharged from hospital B on December 5.

The patient’s hospital course was complicated by respiratory failure that required intubation and ventilation, acute renal failure, pneumothorax and pneumoperitoneum, and anemia and hypotension. Fever resolved 21 days after admission to hospital A. Knee swelling persisted for ≈6 weeks. Antimicrobial therapy administered to the patient while he was an inpatient in hospital B included meropenem, moxifloxacin, vancomycin, ceftazidime, gentamicin, and trimethoprim/sulfamethoxazole. The patient was discharged with oral doxycycline and trimethoprim/sulfamethoxazole to a rehabilitation facility 7 weeks after his initial hospital admission.

Clinical isolates were analyzed to confirm *B. pseudomallei* infection and to determine the genetic origin of the isolate strain. Specimens from hospital A had been destroyed by the time the patient’s melioidosis was diagnosed, which precluded the possibility of determining whether the presumptive *E. coli* infection was actual or misdiagnosed. After receipt at the Arizona State Public Health Laboratory, an isolate was submitted to the Centers for Disease Control and Prevention (Atlanta, GA, USA) for confirmation, and bacterial DNA was extracted and sent to the Translational Genomics Research Institute (Phoenix, AZ, USA) for genetic characterization. Molecular analyses determined that the isolate strain originated from Southeast Asia, most likely Malaysia, or a nearby country. Serology testing performed 6 weeks post infection demonstrated a *B. pseudomallei* indirect hemagglutination assay titer of 160; any titer is considered positive in a person living in an area where the disease is nonendemic (*2*). Serum samples collected early in the course of illness were not available for testing.

The patient and his family were interviewed to determine travel history and possible sources of exposure. No lifetime travel outside of the United States and only limited intrastate and interstate travel were established. The epidemiologic investigation, therefore, focused on the patient’s home and work sites. Possibilities for exposure included occupational exposure to imported vehicle parts, exposure to a person or object from a disease-endemic area, recreational exposure to imported soil or plants, or inoculation with contaminated medication.

Extensive investigation showed no evidence of exposure at the patient’s worksite and no known exposure to any person or objects from a disease-endemic area. We conducted multiple on-site residential investigations, primarily focusing on the patient’s self-reported history of exotic plant repotting. Plant soil and root samples were collected in and around the patient’s home 6 weeks after diagnosis (winter), and 6 months later (summer) and were taken for analysis to the select agent laboratory of Northern Arizona University (Flagstaff, AZ, USA). *B. pseudomallei* could not be cultured from any of the samples tested.

Infection may have occurred from exposure to contaminated medical products. Because the patient was initially hospitalized with sepsis identified as *E. coli*, sepsis might have been the source of his knee pain, and he was subsequently inoculated with *B. pseudomallei* during knee arthrocentesis or from a contaminated oral or intravenous medication. However, investigation of possible medication contamination did not yield any remarkable results.

## Conclusions

Despite extensive investigation, when, how, or where the patient was exposed to *B. pseudomallei* remains unclear. Although travel to a disease-endemic area including Southeast Asia was ruled out, molecular analysis of the etiologic agent showed that it was consistent with Southeast Asian origin.

This case demonstrates the difficulty in diagnosing a disease caused by a rare organism not endemic to the area and the complications that can ensue from delayed diagnosis. Unfortunately, we could not identify the source of exposure despite an aggressive epidemiologic, environmental, and laboratory investigation. Heightened awareness and surveillance by public health officials for this select agent is critical to learning more about the possible presence of *B. pseudomallei* in the United States.
